# Idiosyncratic representation of peripersonal space depends on the success of one's own motor actions, but also the successful actions of others!

**DOI:** 10.1371/journal.pone.0196874

**Published:** 2018-05-17

**Authors:** Yann Coello, François Quesque, Maria-Francesca Gigliotti, Laurent Ott, Jean-Luc Bruyelle

**Affiliations:** Univ. Lille, CNRS, UMR 9193—SCALab—Sciences Cognitives et Sciences Affectives, Lille, France; Universiteit van Amsterdam, NETHERLANDS

## Abstract

Peripersonal space is a multisensory representation of the environment around the body in relation to the motor system, underlying the interactions with the physical and social world. Although changing body properties and social context have been shown to alter the functional processing of space, little is known about how changing the value of objects influences the representation of peripersonal space. In two experiments, we tested the effect of modifying the spatial distribution of reward-yielding targets on manual reaching actions and peripersonal space representation. Before and after performing a target-selection task consisting of manually selecting a set of targets on a touch-screen table, participants performed a two-alternative forced-choice reachability-judgment task. In the target-selection task, half of the targets were associated with a reward (change of colour from grey to green, providing 1 point), the other half being associated with no reward (change of colour from grey to red, providing no point). In Experiment 1, the target-selection task was performed individually with the aim of maximizing the point count, and the distribution of the reward-yielding targets was either 50%, 25% or 75% in the proximal and distal spaces. In Experiment 2, the target-selection task was performed in a social context involving cooperation between two participants to maximize the point count, and the distribution of the reward-yielding targets was 50% in the proximal and distal spaces. Results showed that changing the distribution of the reward-yielding targets or introducing the social context modified concurrently the amplitude of self-generated manual reaching actions and the representation of peripersonal space. Moreover, a decrease of the amplitude of manual reaching actions caused a reduction of peripersonal space when resulting from the distribution of reward-yielding targets, while this effect was not observed in a social interaction context. In that case, the decreased amplitude of manual reaching actions was accompanied by an increase of peripersonal space representation, which was not due to the mere presence of a confederate (control experiment). We conclude that reward-dependent modulation of objects values in the environment modifies the representation of peripersonal space, when resulting from either self-generated motor actions or observation of motor actions performed by a confederate.

## Introduction

To ensure appropriate and safe interactions with our physical and social environment, at every moment our brain needs to isolate from the flow of sensory information the stimuli that are of interest with respect to the current situation. In particular, objects present in the space immediately surrounding the body receive particular attention because this is where most of the interactions with the environment take place. Numerous studies have demonstrated that objects within reach are coded not only on the basis of their visual features, but also in motor terms so as to anticipate deployable actions and their effects [1, 2]. Accordingly, the brain retains a segmented representation of the external world, dissociating the peripersonal and extrapersonal spaces [3–6], depending on whether objects are or are not relevant for impending motor actions. The concept of peripersonal space, initially proposed to account for the multimodal coding of action space [7], was later used to also account for the protective buffer surrounding the body [8–10], although the two assumptions are not mutually exclusive [5, 11]. In support of the segmented representation of space, objects in peripersonal space activate a dorsal network linking the dorsal occipital cortex to parietal and motor frontal areas, whereas objects in extrapersonal space activate a ventral network linking the ventral occipital cortex to the temporal and non-motor frontal areas [12–14]. As a result of this functional neural organisation, significantly faster visual processing, as indexed by a consistent N1 visual component with faster latencies and greater amplitudes in the visual electroencephalography (EEG) signals, as well as enhanced parietal activity was observed for objects located in near space compared to far space. This was interpreted as an attentional “prior-entry” effect [15] accelerating the processing of objects that appear within reachable space in order to facilitate a rapid interaction with them [16]. In the same vein, neuropsychology cases revealed attentional deficits resulting from brain damage in the parieto-temporal regions that were predominantly observed in either the near space [17–18] or far space [19–20].

Dominant theoretical frameworks emphasize that peripersonal space is a dynamic representation [21–22], within which object coding specifically involves a multisensory [7, 23] and body-part centred frame of reference in relation to the action system [3, 5, 24]. In agreement with this view, several studies have demonstrated that altering arm length in the body schema through tool-use [25], or biasing the spatial outcome of a manual reaching action [21] modifies the representation of peripersonal space. Accordingly, stroke patients with brain damage in motor-related regions showed specific deficits concurrently in visually controlled motor actions and in the representation of peripersonal space [26–27].

Interestingly, peripersonal space seems also to serve as spatial reference independently of the presence of manipulable objects in the environment. As evidence, Quesque, Ruggiero, Mouta, Santos, Iachini and Coello [28] demonstrated that enlarging the peripersonal space by using tools increases the interpersonal crossing distance maintained during navigation. From this finding, Coello & Iachini [24] extended the concept of peripersonal space to embrace the fact that it not only contains the objects and hazards that the organism must consider when interacting with the surrounding environment, but it also specifies a safe and private area for interacting with conspecifics in social contexts (see also [10, 29–30]). In agreement with this view, individuals with enlarged self-representation of peripersonal space reported higher rates of social anxiety [31–32] and phobia [33], sometimes associated with claustrophobic fear [34]. These results are thus consistent with a motor function but also a defensive function for the peripersonal space [35], contributing then to the organisation of object-directed actions but also to the regulation of social life [24]. They also suggest that significant overestimation of one's peripersonal space may play an important role in the development of anxiety disorders [3, 32, 36–37].

Although a wealth of data is available relating to the dynamic feature of peripersonal space and its implication in object coding, both in social and non-social contexts, how the representation of peripersonal space is altered by the perceived value (i.e. reward) of objects in the environment is still largely unknown. Every object that we perceive has a positive/negative relationship to motor action depending on previous experiences and outcomes [38–39]. In any circumstances, object value is automatically perceived and incorporated into the mental representations of the objects, contributing then to connecting vision to behaviour [40]. The presence of objects with positive/negative value thus influences how we think about and react to the world around us [41]. For instance, the close proximity of threatening objects (e.g., dangerous tools) induces shrinking of peripersonal space such that motor actions towards these stimuli are restrained [42]. However, how the value of objects in the environment shapes the representation of peripersonal space in the absence of obvious threats is not fully understood. If the probability of getting a better action outcome implies selecting an object to the left rather than to the right, one might expect our representation of peripersonal space to be distorted with an increased saliency of the left region of space. Although this issue has not been specifically addressed, studies on visual perception have shown that neural activity in the visual cortex is modulated by the behavioural significance of stimuli [16]. For instance, in a task requiring detection of visual similarity with a previously learned visual pattern (reward contingent stimulus), modulatory effects in primary visual areas were observed for the reward-yielding visual stimuli [43]. This suggests an influence of higher-order brain regions on low-level feature processing [16]. This influence was also observed when the relevance of the visual stimuli was contingent on action [44]. We can therefore expect the action-dependent value of visual stimuli to alter not only low-level sensory processing and motor behaviour, but also the representation of peripersonal space.

The issue of the value of visual stimuli in relation to motor action becomes even more important when considering social contexts. The selection of appropriate objects for action when other people are close to us can determine the effectiveness of social interaction and cooperation [45]. Furthermore, observing the consequence of someone else's action can influence our own evaluation of objects of interest in the environment [46]. Thus, the social context and the observation of other individuals’ actions are in principle likely to alter the representation of both the value of visual stimuli and the representation of peripersonal space. In agreement with this, Fujii, Hihara and Iriki [47] showed, in a monkey study, that when the monkeys sat near each other but did not interact, each monkey’s parietal activity showed robust response preference for actions towards both right and left stimuli present in their respective peripersonal spaces. When the location of monkeys enabled them to reach for the same food item, parietal cortex adapted its response properties by discarding some stimuli and recruiting different neural populations, including a modulation of the neuronal activity in the pre-frontal cortex [48]. This observation suggests that the same object can be included or not in the peripersonal space, depending on its value and the social context. Furthermore, the pre-frontal cortex appears as a key cerebral region in relation to the parietal cortex for modulating the representation of peripersonal space.

So far, no human study has specifically investigated the effect of changing the value of visual objects on the representation of peripersonal space, when interacting with these objects or observing someone else interacting with them. This issue represents the purpose of our study. The task for the participants consisted in selecting with the hand a number of visual targets in the peripersonal space with the aim of gaining a positive outcome (reward contingent stimuli). The probability that a selected target yielded positive outcome (change of colour from grey to green) was spatially manipulated so that it was unexpectedly higher in the participant's proximal or distal space. In two experiments, we tested the effect of changing the spatial distribution of targets yielding positive outcomes on a manual reaching action and on peripersonal space representation, when the target-selection task was performed in either an isolated or a social interaction context.

## Experiment 1

Participants were engaged in a two-alternative forced-choice reachability-judgment task, before and after having performed a target-selection task. In the latter, participants had to maximize successful manual reaching actions by selecting out of the set of 32 randomly displayed targets, 10 targets yielding a positive outcome (i.e. reward). The probability that the target selected was a reward-yielding target was the same in all experimental conditions (50%), except that the spatial distribution of reward-yielding targets was covertly manipulated, being either 75% in the proximal space, 75% in the distal space, or 50% in both spaces.

## Materials and methods

### Participants

Sixty participants, aged 18–35 (mean age: 23.31, SD: 4.76), gave written informed consent prior to inclusion in the experiment. They were all right-handed (as determined by the Edinburgh Handedness Inventory [49]; mean score: 0.85, SD: 0.24), and had normal or corrected-to-normal vision. They had no prior knowledge of the scientific purpose of the study. The protocol received approval by the Institutional Ethics Committee (Ref. Number 2017-7-S52) and conformed to the principles of the Declaration of Helsinki [50].

### Materials and stimuli

**Fig 1:**
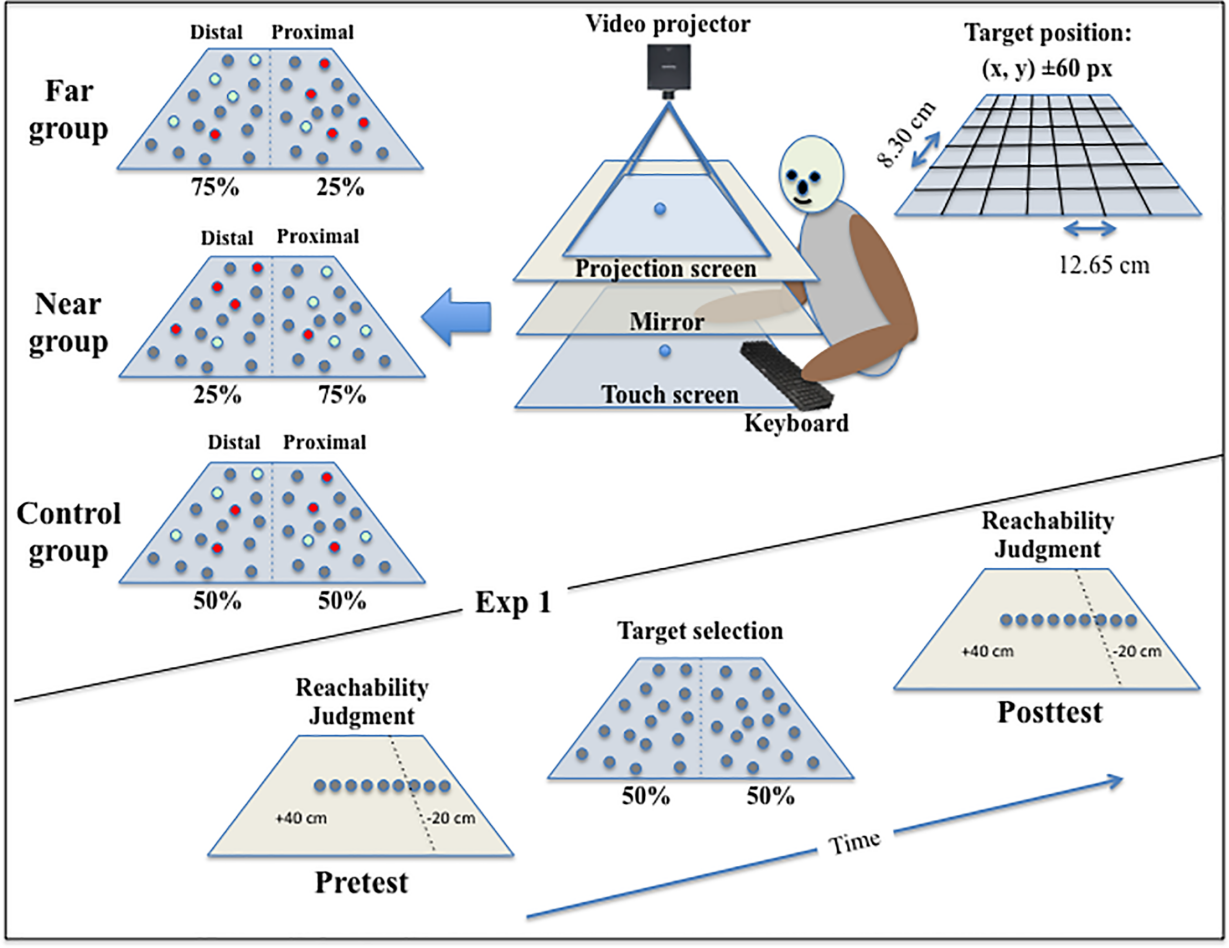
Upper part: Sketch of the apparatus and stimuli display in the target-selection and the reachability-judgment tasks of Exp. 1. Left: The probability of selecting a reward-yielding target was 50%, 25% or 75% in the proximal and distal spaces. Centre: In the target-selection task, the 32 targets were directly visible on the touch-screen table. In the reachability-judgment task, the 31 targets were visible through the mirror as displayed by the video projector. Right: the position of each target displayed (32 out of 42 possible locations separated by 12.65 x 8.30 cm) was randomly computed from the centre of the cell ± 60 pixels). Lower part: Timeline for the tasks.

A schematic representation of the apparatus is presented in Fig 1. Participants were seated in front of a horizontal 40” touch-screen table (Samsung SUR40). Depending on the task, they were told to process information displayed on the touch-screen table or resulting from the projection of a video projector placed above it.

#### Reachability-judgment task

In the reachability-judgment task, the apparatus consisted of a video projector (Infocus 3926D) positioned 79 cm above a 200 × 150 cm horizontal translucent screen and projecting a 161 x 118 cm image onto it. The translucent screen was positioned 66 cm above the touch-screen table (109.5 x 70.74 cm), which was placed at 72.8 cm from the floor. A mirror positioned halfway between the translucent screen and the touch-screen table projected the visual scene displayed by the video projector at the level of the touch-screen table, but hiding the table from direct view. Thirty-one visual targets, ranging from -20 cm to +40 cm (inter-target distance 2 cm) relative to the participant's actual maximum reach were each presented for a duration of 100 ms along the mid-sagittal axis of the participants’ body and with an inter-stimulus interval of 3 seconds. The participants’ task was to judge, for each target, if it was at a reachable distance or not. Their responses were recorded using a computer keyboard placed on the left side of the touch-screen table, thus under the mirror.

#### Target-selection task

In the target-selection task, the projector was turned off and the mirror was removed, thus offering a direct view of the touch-screen table. Thirty-two grey circle targets (diameter 2.7 cm) were randomly displayed on the black background of the 40” touch-screen table (active area of 1920 x1080 pixels, 88.56 x 49.81 cm) according to a non-visible distribution grid (Fig 1). The latter was composed of 42 cells (6 rows x 7 columns) that covered the whole touch-screen table. When positioned in the centre of the cells, the inter-target distance was 12.65 cm (274 px) along the x axis and 8.30 cm (180 px) along the y axis. On each block of manual reaching actions, the 32 grey targets were displayed at random locations (from 0 to 60 pixels from the centre of the cells in all directions) in randomly selected cells, thus leaving 10 cells empty. The configuration of the set of targets changed in each block, which gave the impression of a random distribution. Targets were selected by touching them on the screen with the right index finger, with the consequence that the touched target changed its colour. If it turned to green (50% of the targets), a sound of clinking coins was played and the participants gained one point. If the selected target turned to red (50% of the targets), a buzzing sound was played and the participants gained no point. On each block, participants had to freely select 10 targets trying to maximize the point count by selecting the reward-yielding targets. When the tenth target was selected, a new configuration was displayed and the target-selection task started again until 40 blocks were completed.

The probability that a selected reward-yielding target depended on the location of the target on the touch-screen table and the group assignment. In the Control group (N = 20), the probability to select a reward-yielding target was 50% in both the near (3 proximal rows) and far (3 distal rows) space. In the Far group (N = 20), the probability to select a reward-yielding target was 25% in the near space and 75% in the far space. On the contrary, in the Near group (N = 20), the probability to select a reward-yielding target was 75% in the near space and 25% in the far space. Participants were unaware of their group assignment.

### Procedure

To begin with, the participants completed the written consent and the Edinburgh Handedness Inventory [49] and were then seated in front of the touch-screen table in a dark room. Next, they were randomly assigned to one of the three experimental groups (Far, Control, Near). Then, the experimenter measured their arm length, inter-shoulder distance and eye-level height relative to the touch-screen table and entered these measures into a customised computer program that computed the visual stimuli to be used. Following this step, instructions relating to the two tasks were provided, and participants then performed successively the reachability-judgment task (pretest), the target-selection task, and for the second time the reachability-judgment task (posttest). During the reachability-judgment task, participants were seated with their right hand resting on the touch-screen table in front of their chest. Their left hand was positioned on the left side of the touch-screen table on a computer keyboard, with their left middle and index fingers respectively placed on the left and right keyboard arrow keys. Each time a target was presented and seen through the mirror, participants provided their response by pressing one of the two keyboard keys (e.g. left for “non-reachable” and right for “reachable”, counterbalanced across participants). Each of the 31 target locations was presented 4 times, in a random order, yielding a total of 124 reachability judgments.

Following the first reachability-judgment task (pretest), the mirror was removed, allowing participants to see their hands and the touch-screen table. They were then instructed to select in each block 10 targets out of the 32 presented at random locations, and to maximize the point count by selecting the reward-yielding targets (providing thus one point). In total, they performed 40 blocks of manual reaching actions yielding 400 successive target selections. A counter visible in the proximal part of the touch screen provided real-time updating of point count. Following this task, a second reachability-judgment task (posttest) was performed. Finally, a post-experiment debriefing was administered to assess whether participants were aware of the hypotheses tested. None of them were. Only a few participants have conjectured that a spatial rule determined the location of the reward-yielding targets, but none could articulate the rule. In all, the experimental session lasted approximately 45 minutes.

### Data and analysis

Reachability estimates made for the 31 targets were analysed separately for the pretest and posttest, with all estimates being corrected for actual arm length. The boundary of peripersonal space was determined using a maximum likelihood fit based on the second-order derivatives (quasi-Newton method) to obtain the logit regression model that best fitted the reachable/unreachable responses (see [21] for details). The logit regression model was defined by the equation: y = e ^(α ± β X)^/ (1+ e ^(α ± β X)^), in which y was the (reachable, unreachable) response, X was the crossing distance, and (–α/β) was the critical value of X corresponding to the transition between reachable and unreachable stimuli, thus expressing the perceived boundary of peripersonal space. The goodness of fit in logistic regression was estimated through R coefficient to check how well the model fits the data. The discrimination threshold was defined as the distance between the critical value of X at which the transition from one type of response to the other type of response occurred, and the critical value of X at which the probability associated with the logit function was .84 (see [51]), which was computed as: [Log (0.84/(1–0.84))/β]–[Log(0.5/(1–0.5))/β]. A smaller discrimination threshold indicated an easier separation between reachable and unreachable responses.

We also analysed reaction time in the reachability-judgment task for the 5 proximal targets, the 5 distal targets and the 5 targets at the boundary of peripersonal space. Reaction time corresponded to the time elapsed from the presentation of the stimulus to the keyboard key press. The boundary of peripersonal space and the discrimination threshold were analysed using repeated measures ANOVA on posttest-pretest scores for the three groups (Far, Control, Near). The goodness of fit in logistic regression was analysed in the pretest and the postest using Group (Far, Control, Near) ANOVAs. Reaction time was analysed using a Session (Pretest, Posttest) × Space (Peripersonal, Boundary, Extrapersonal) × Group (Far, Control, Near) ANOVA, with repeated measures on the first two factors.

Concerning the target-selection task, the analysis first focused on the location of the targets selected across the manual reaching actions. This was done by plotting the number of times each target location was selected on the touch-screen table, relative to the total number of targets (density map). For that purpose, we pooled the data from the two proximal rows (1 and 2), the two central rows (3 and 4) and the two distal rows (5 and 6). Statistical analysis was performed using a Group (Far, Control, Near) × Space (Proximal, Central, Distal) Chi-squared test. Second, in order to precisely investigate when the targets selection in the proximal and distal spaces diverged depending on the group, we computed the number of times the participants selected a target, as well as the number of successful actions (reward-yielding targets), in the proximal hemi-space (rows 1, 2, 3) and the distal hemi-space (rows 4, 5, 6). Statistical analysis was performed on the number of targets (or reward-yielding target) selected in the proximal hemi-space using a Group (Far, Control, Near) × Block (1 to 40) ANOVA with repeated measures on Block. Finally, in order to evaluate the spatial effect of changing targets selection strategy across groups, we computed the mean amplitude of manual reaching actions in the different blocks and statistically compared the first blocks (2 and 3) to the last blocks (39 and 40) through a Groups (Far, Control, Near) × Block (First, Last) ANOVA, with repeated measures on Block (the first block was considered as practice block).

For all analyses, distribution normality was tested using the Shapiro-Wilk test, and homogeneity of the covariance matrices was tested using the Box's M test. In case of lack of normality of the data distribution, non-parametric test was used to compare groups (Kruskal-Wallis ANOVA). In case of violation of the sphericity assumption (i.e., epsilon < 1), Huynh-Feldt adjustments to the p-values were reported. Effect sizes were indexed using partial Eta-squared (η^2^_p_). Post-hoc comparisons were performed using *Tukey's* HSD test (α = .05 for all comparisons).

## Results

### Reachability estimates

**Fig 2:**
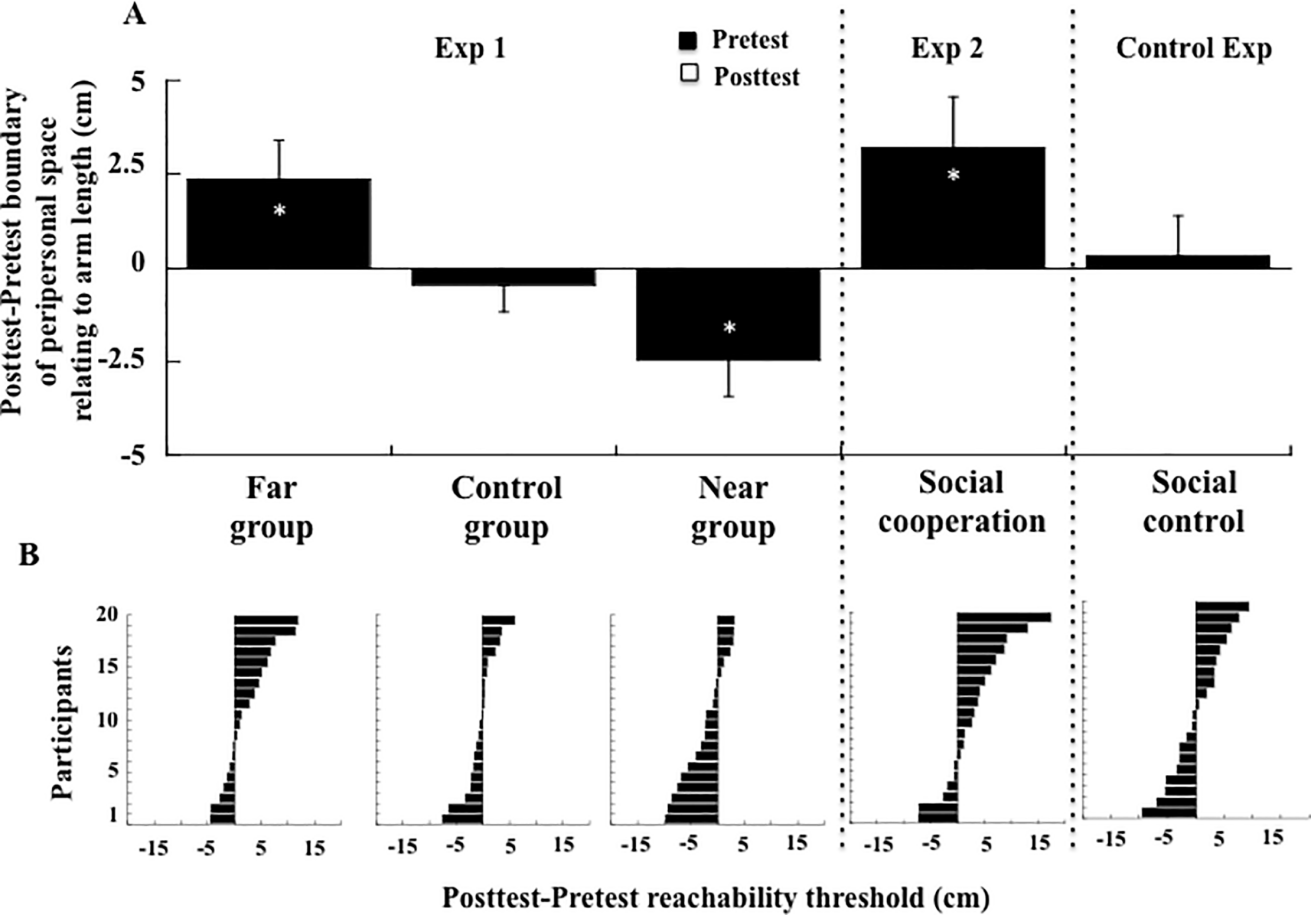
(A) Posttest-Pretest boundary of peripersonal space relating to arm length (cm) as a function of the group in Exp. 1 (Far group, Control group, Near group), in Exp. 2 (social) and in the control experiment. Stars indicate significant difference between the pretest and the posttest. (B) Individual variation of performance between the pretest and the posttest (negative and positive signs indicate respectively reduction and expansion of peripersonal space).

In the pretest, the mean perceived boundary of peripersonal space overestimated the actual maximum reachability by 6.22 cm on average, which corresponded to an overestimation of arm length by 8.48% (average arm length = 73.28 cm). The two-way ANOVA on reachability estimates revealed a significant Session (Pretest, Postest) × Group (Far, Control, Near) interaction (F(2, 57) = 7.04, p = .002, η^2^η^2^_p_ = 0.20). Post-hoc comparisons showed that in the posttest, compared with the pretest, the Near group had a shorter peripersonal space (-2.49 cm, p = 0.02; with for pretest: 6.74 cm and postest: 4.25 cm) while the Far group had a larger peripersonal space (+2.35 cm, p = 0.03; with for pretest: 6.55 cm and postest: 8.90 cm). By contrast, the Control group's overall change was small and not significant (-0.46 cm, p = .61; with for pretest: 5.35 cm and postest: 4.89 cm). Within the Near and Far groups combined, 26 of the 40 participants (65%) individually showed their group's directional bias (see individual results, Fig 2). Concerning the goodness of fit in logistic regressions, R coefficient (0.797) on average) was found to be not different in the pretest when comparing the Near (0.783), the Far (0.745) and the Control group (0.796; Kruskal-Wallis H (2, 60) = 2.46, p = .30). In the posttest, R coefficient was instead lower in the Far (0.767) compared to the Near (0.842) and Control group (0.848; Kruskal-Wallis H (2, 60) = 10.44, p<0.01). The discrimination threshold was 7.88 cm on average. It was smaller in the posttest (6.80 cm) compared to the pretest (8.96 cm; F(1, 57) = 9.48, p < .01, η^2^p = .14), but similarly in the three groups (F(2, 57) = 0.86, p = .43), which did not differ (F(2, 57) = 2.16 p = .12).

Analysis of reaction time (577 ms on average, see Table 1) showed an interaction between Space (Peripersonal, Boundary, Extrapersonal) and Session (Pretest, Posttest), (F(2,57) = 7.99, p < .01, η^2^p = .12). Post hoc analyses revealed that reaction time in the posttest decreased compared to the pretest for targets in peripersonal (-63 ms) and extrapersonal (-53 ms) spaces (both p < .001), but not for targets located at the boundary of peripersonal space (-15 ms, p = .11). Moreover, this interaction was not modulated by the Group (F(2,114) = 1.62, p = .17), despite the fact that the Far group (522 ms) showed on average a slightly faster reaction time than the two other groups (Near group: 604 ms, Control group: 613 ms; F (2, 57) = 4.12, p = .02, η^2^_p_ = 0.13). For the Far group, the lower goodness of fit found in the logistic regressions in posttest might thus result from the slightly faster reaction times observed in this group.

**Table 1:** Mean reaction time (SD in brackets) in the reachability-judgment task of Exp 1.

Group	Session	Peripersonal	Boundary	Extrapersonal
**Far**	Pretest	468 (99)	596 (127)	555 (100)
	Posttest	409 (102)	576 (101)	528 (88)
**Control**	Pretest	574 (149)	694 (164)	638 (144)
	Posttest	499 (133)	697 (158)	579 (94)
**Near**	Pretest	511 (116)	719 (195)	660 (163)
	Posttest	458 (102)	691 (168)	586 (120)

### Target-selection task

**Fig 3:**
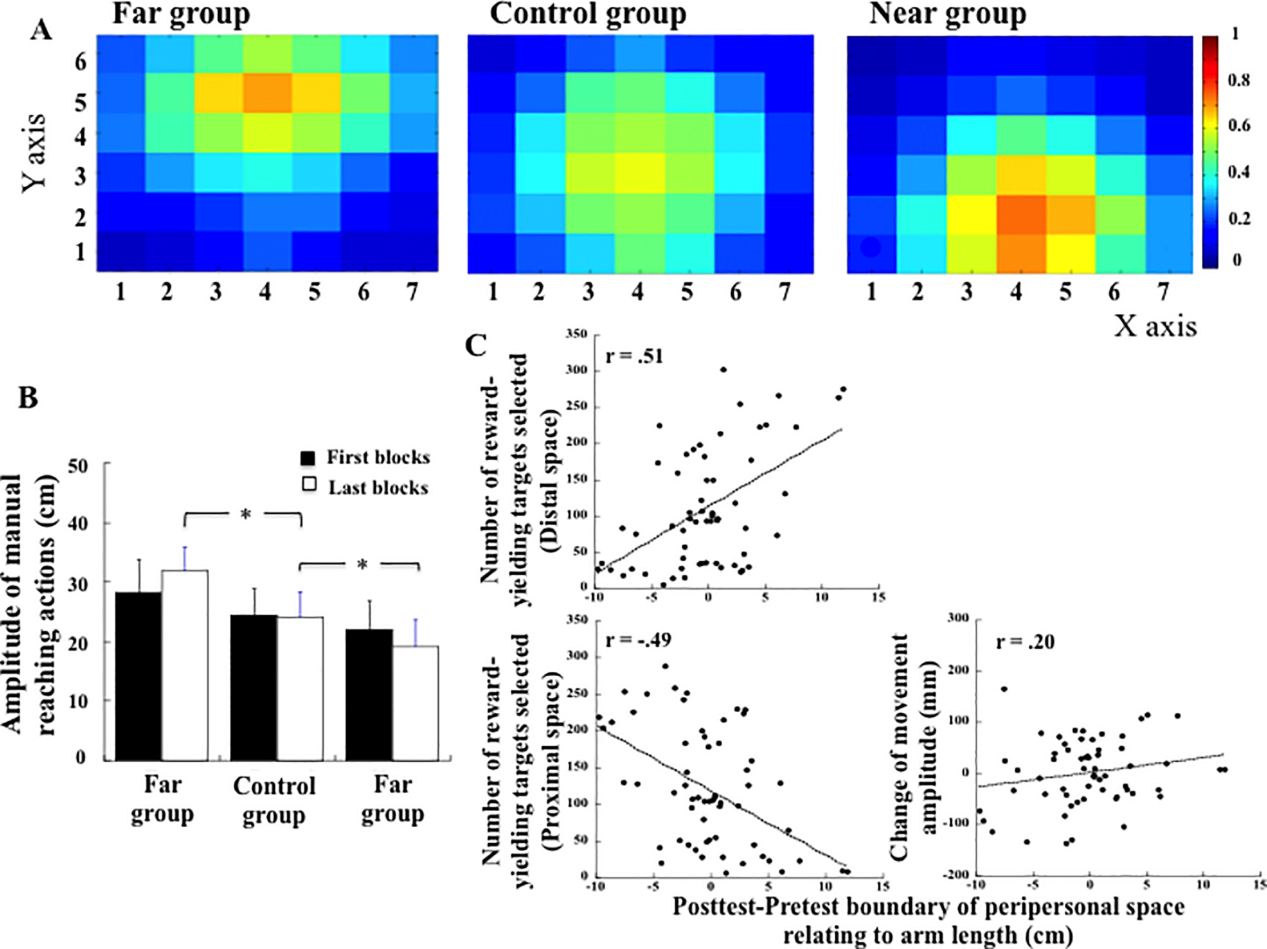
(A) Density maps of the target selected in the target-selection task by all the participants across the 400 manual reaching actions. Blue colour indicates infrequent selection; Red colour indicates frequent selection. (B) Mean amplitude of manual reaching actions in the first and last blocks for the Far group, Control group and Near group (Exp. 1). Stars indicate significant differences. (C) Linear regression (individual data) between the number of reward-yielding targets selected in the distal space and the change of reachability threshold (upper-left), between the number of reward-yielding targets selected in the proximal space and the change of reachability threshold (lower-left), and between the change of movement amplitude (mm) and the change of reachability threshold (right). The linear regression coefficients are indicated in the upper-left part of each plot.

The amplitude of manual reaching actions across the 400 target selections was on average 25.3 cm, the length of the touch-screen table being 50 cm. Comparing the targets selected in the proximal (rows 1 and 2), central (rows 3 and 4) and distal (rows 5 and 6) spaces, we observed that the frequency of target selection differed across the three groups (χ^2^(4) = 3211, p < .001). Among the 8000 targets selected (10 targets x 40 blocks x 20 participants), the Far group selected 1284 targets (16%) in the proximal space and 3719 targets (46%) in the distal space. The Near Group selected 4136 targets (52%) in the proximal space and 1284 targets (16%) in the distal space. The Control group selected 2719 targets (34%) in the proximal space and 2005 targets (25%) in the distal space. The density maps presented in Fig 3A describe the three groups' respective distributions of the selected targets.

The mean amplitude of manual reaching actions across the 400 target selections was 31.0 cm from the edge of the touch-screen table in the Far group, 24.8 cm in the Control group, and 20.7 cm in the Near group. When comparing the first blocks to the last blocks (Fig 3B), we found a significant Group (Far, Control, Near) × Block (First, Last) interaction (F(2, 57) = 5.85, p = .004; η^2^_p_ = 0.17). Post hoc comparisons showed that the Far and Near groups did not differ from the Control group in the first blocks (respectively, p = .09 and p = .65, with respectively 28.2 cm, 22 cm, and 24.3 cm), but did differ in the last blocks (respectively 31.9 cm, 19.1 cm and 24.1 cm). The Far group selected targets at a further distance (p < .01) and the Near group selected targets at a closer distance (p = .01) than the Control group (Fig 3B).

**Fig 4:**
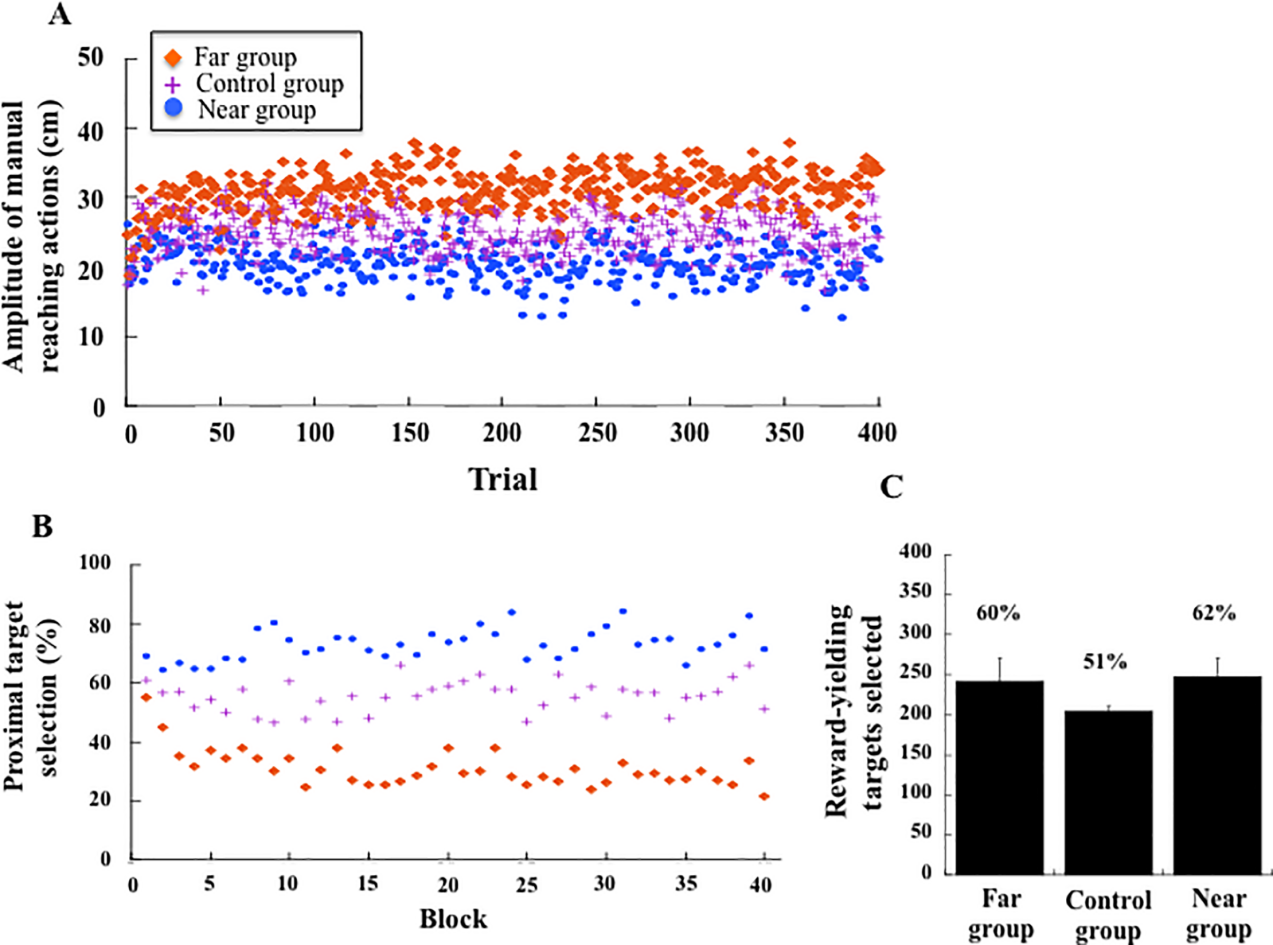
(A) Mean amplitude of manual reaching actions in the target-selection task across the 400 target selections (Exp. 1) for the Far group, Control group and Near group. (B) Mean percentage of target selected in the proximal space (row 1, 2, 3) across the 40 blocks. The three groups diverged from the 3^rd^ block. (C) Mean number and percentage of reward-yielding targets selected in the Far group, Control group and Near group.

When analysing the variation of target selection across blocks, we observed that the change in target selection appeared early in the experimental session (Fig 4A). In order to determine when the groups diverged in terms of targets selection, we computed the number of times targets were selected in the proximal hemi-space (rows 1, 2, 3) compared to the distal hemi-space (rows 4, 5, 6), for each participant and each group across the blocks (Fig 4B). Analysis of variance revealed an interaction between the Group and the Block (F(78, 2223) = 1.67, p < .001, η^2^_p_ = 0.055). Post hoc comparisons showed that the Far group selected more targets in the distal space and diverged from the Near group starting in the 3^rd^ block (p = .04). As a consequence, and in agreement with the design of the experiment, we observed that the percentage of reward-yielding targets selected differed according to the group (Fig 4C). The Near and Far groups selected more reward-yielding targets (respectively 60% and 62%) compared to the Control group (51%, F(2, 57) = 21.65, p < .001, η^2^_p_ = 0.43).

### Relation between targets selection, movement amplitude and peripersonal space

First, in order to test whether the change of peripersonal space representation was related to the amount of rewards obtained, we computed the linear regression between the number of reward-yielding targets selected in either the proximal or the distal space and the change of peripersonal space, considering all participants and all groups. As shown in Fig 3C, the linear regression coefficient (R) between the two variables was equal to -0.49 when considering the reward-yielding targets selected in the proximal space, while it was equal to 0.51 when considering the reward-yielding targets selected in the distal space (both p < .05).

Second, in order to test whether the change of peripersonal space representation was related to the motor performances, we computed the linear regression between the change in the amplitude of manual reaching actions through the target selection task and the change of peripersonal space, considering all participants and all groups. As shown in Fig 3C, the linear regression coefficient (R) between the two variables was equal to 0.20 (p = .12).

## Discussion

The aim of Experiment 1 was to assess the effect of selecting targets yielding a positive outcome (reward-yielding targets) on manual reaching actions and the representation of peripersonal space, when the probability of selecting those targets was spatially biased towards the proximal or distal space. In the Far group, the probabilities of selecting a reward-yielding target were respectively 75% and 25% in the distal and proximal spaces. In the Near group, these probabilities were 25% and 75% in the distal and proximal spaces, respectively. In the Control group, the probability of selecting a reward-yielding target was 50% in both spaces.

The first important result of this experiment was that the targets selected by the participants throughout the experiment were dependent on the spatial distribution of the reward-yielding targets. Indeed, whereas all targets in the workspace were selected by the participants across the blocks, the rate of target selection at a particular location was certainly affected by the probability that a reward-yielding target was at that location. The Far group selected 46% of the targets in the distal space and 16% in the proximal space; the Near group selected 16% of the targets in the distal space and 52% in the proximal space; and the Control group selected 34% of the targets in the distal space and 25% in the proximal space. The Far, Near and Control groups selected approximately the same number of targets in the central space (38%, 32%, 41%, respectively). The average amplitude of manual reaching actions towards targets was thus broader in the Far (31 cm from the edge of the touch-screen table) than the Near (21 cm) group, the Control group falling in-between (25 cm). The analysis of performances across the blocks showed that the selection of targets diverged among the groups starting in the 3^rd^ block, i.e. after the completion of about 20 target selections. Adaptation of the groups to the spatial distribution of reward-yielding targets was significant since the change in the amplitude of manual reaching actions between the first and last blocks was 3.7 cm in the Far group and -2.9 cm in the Near group, whereas it was only 0.2 cm in the Control group. Hence, participants seemed sensitive to the probability of performing a successful action depending on the locations of the targets, although this remained out of awareness. The changes thus reflected implicit learning, and this expands the findings of previous studies on attention [52–54] and ocular control [38, 55], to object-oriented manual actions.

The second important result of this experiment is that the representation of peripersonal space was influenced by the spatial distribution of the reward-yielding targets and the resulting change in the manual reaching actions. Although participants slightly overestimated their peripersonal space, as indexed by the reachability performances (8.48% of arm length on average), which agrees with previous studies [24, 56], this overestimation was modulated by the target-selection task. Peripersonal space increased in the Far group (2.35 cm), whereas it shrank in the Near group (- 2.49 cm). This result indicates that the implicit detection of the distribution of reward-yielding targets in the target selection task, and associated change in the manual reaching actions, modified the representation of peripersonal space. When the distribution of reward-yielding targets was not biased, the extent of peripersonal space did not change significantly (-0.46 cm). This suggests that the modification of the representation of peripersonal space in the Far and Near groups cannot simply be attributed to the series of motor actions performed, but resulted rather from the distribution of reward-yielding targets and associated manual reaching actions. According to our hypothesis and as shown by the correlation analysis (testing the relation between the reward-yielding targets location or reaching actions and the extent of peripersonal space), the change of peripersonal space representation appears however more related to the distribution of the selected reward-yielding targets in the proximal and distal spaces, than to the change of movement amplitude. This suggests that the representation of peripersonal space is highly sensitive to objects’ value in the environment, and not only to the motor actions performed towards these objects.

The accuracy of the reachability judgements, as indexed by the discrimination threshold [51], was not different in the three groups (on average 13 cm), suggesting that the difficulty of the task was equivalent whatever the distribution of the reward-yielding targets. It was also not influenced by the task repetition, which suggests that task difficulty did not change over time. Furthermore, reaction time (577 ms) was shorter for the near and far targets compared to the targets at the boundary of reachable space in all groups. This effect echoed findings reported previously [21, 26, 57], and highlighted the increased requirement for motor-related information to achieve the reachability decision for targets presented at the boundary of reachable space [57–58]. The repetition of the reachability-judgment task in the posttest led, however, to a facilitation effect signified by shorter reaction times, although only for near and far targets. This indicates that judging targets at the boundary of reachable space remained difficult even when the task was thoroughly familiar, and led to a strong interaction between multisensory and motor information, as suggested in previous fMRI [26, 57] and TMS [58] studies.

As a whole, the results of Experiment 1 reveal that the value of the stimuli in the environment influences not only self-selected manual reaching actions, but also the representation of peripersonal space. Recently, Coello et al. [42] showed that presenting dangerous objects led to a reduction of peripersonal space, but only when the threatening part of the dangerous objects was oriented towards the participant, as compared to when it was oriented safely away. This suggested that the interpretation of the higher-order context in reference to the body is crucial in specifying peripersonal space, considered as a defensive space [24]. The present study extends these findings by showing that non-threatening stimuli also influence the representation of peripersonal space. Indeed, by manipulating the expected value of visual stimuli depending on their location, we observed a concomitant effect of the distribution of these visual stimuli on the direction of manual reaching actions and the representation of peripersonal space. Thus, the resizing of peripersonal space appears to depend on both bottom-up and top-down factors. For a particular visual stimulus, this implies combining multisensory and action-related information with the value attributed to that particular stimulus, built on previous experience and depending on the context. This is in line with the observation that positive objects trigger a larger response in the visual cortical regions than neutral objects [16, 59], suggesting a functional connectivity between frontal and occipito-parietal areas [60] contributing to perceptual facilitation and attentional control [61]. Accordingly, perceiving the value of a particular visual stimulus influences how we think about and react to the world around us [40], including how we functionally segment the external world in relation to action [62] and how we estimate distances [63].

Although a positive outcome associated with a particular action usually depends on one's own action system, it is sometimes the case that observing actions performed by a confederate also influences the observer's representation of peripersonal space (e.g, [64]). In Experiment 2 we tested the effect of sharing the same working space with a confederate on peripersonal space representation, in a target-selection task implying social interactions.

## Experiment 2

In this experiment, we tested whether the selection of reward-yielding visual targets influenced manual reaching action and the representation of peripersonal space in a similar way than when the target selection task was performed by two participants facing each other. According to ideomotor theories, observing another person’s action activates the same representations in the observer’s cognitive system as usually employed to produce an action on his/her own [65–67]. Assuming that observed motor actions are interpreted through the observer's motor repertoire [68] and that the spatial distribution of reward-yielding visual targets in our target selection task modulates the representation of peripersonal space (Exp. 1), one may expect that observing a confederate performing the target-selection task would modulate the observer's representation of peripersonal space just as though the participant was the actor. In the present experiment, two participants were simultaneously engaged in a two-alternative forced-choice reachability-judgment task, before and after having performed a target-selection task cooperatively. In the latter task, the two participants had to maximize successful manual reaching actions by selecting, out of the set of 32 displayed targets, 12 (6 each) that were expected to be reward-yielding. The spatial distribution of reward-yielding targets was as in the Control condition of Exp. 1, i.e. 50% in both the proximal and the distal spaces.

## Materials and methods

### Participants

Twenty participants, aged 18–50 years (mean age: 29.25, SD: 10.19), gave written informed consent prior to inclusion in the experiment. They were all right-handed (as determined by the Edinburgh Handedness Inventory [49], mean score: 0.86, SD: 0.28), and had normal or corrected-to-normal vision. They had no prior knowledge about the scientific aim of the study. The protocol received approval by the Institutional Ethics Committee (Ref. Number 2017-7-S52) and conformed to the principles of the Declaration of Helsinki [50].

### Materials and stimuli

**Fig 5:**
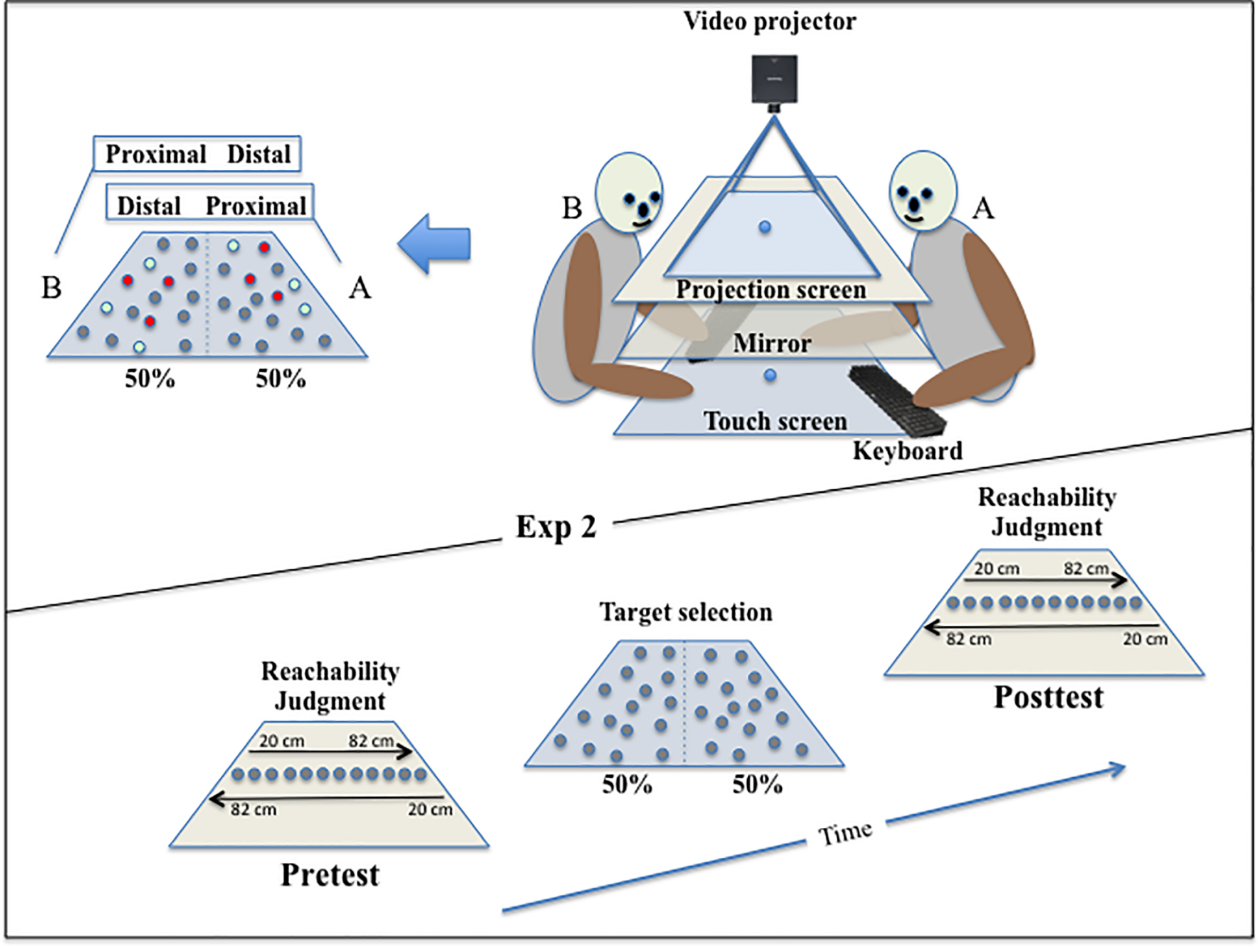
Upper part: Sketch of the apparatus and stimuli display in the target-selection and the reachability-judgment tasks (Exp. 2). Left: The probability of selecting a reward-yielding target was 50% in the proximal and distal spaces. Right: Two participants were facing each other (position A and B). In the target-selection task, the 32 targets were directly visible on the touch-screen table. In the reachability-judgment task, the 31 targets were visible through the mirror projecting the image displayed by the video projector on the touch-screen table. Lower part: Time sequence of the tasks presentation.

A schematic representation of the apparatus is depicted in Fig 5. Two participants were seated facing each other across the touch-screen table. They were told to process information displayed on the touch-screen table or resulting from the projection of a video projector placed above it, depending on the task.

#### Reachability-judgment task

In the reachability-judgment task, the apparatus used in Exp. 2 was identical to that in Exp. 1 except for the following items: Thirty-one visual targets, ranging from 20 cm to 82 cm away from the head position of the two participants (inter-target distance of 2 cm) were each presented for a duration of 100 ms along the mid-sagittal axis of participant’s body and with an inter-stimulus interval of 3 seconds. The participants recorded their (yes-no) responses simultaneously using one of the two external USB keyboards (one for each participant) placed on the left side (relative to each participant) of the touch-screen table, thus under the mirror.

#### Target-selection task

The target-selection task was identical to the one performed in Exp. 1 except for the following items: On each block, the two participants took turns selecting the targets with their right index finger, 6 targets each, for a total of 12 targets out of the 32 grey circle targets randomly displayed on the touch-screen table. Each target turned either green or red immediately after being touched (triggering the associated sound). When the twelfth target had been selected, a new configuration was displayed and the target-selection task started again until 34 blocks were completed. On each block, the two participants had to maximize the point count and the common cumulative number of points obtained was displayed throughout the experiment for the participants on a digital counter located on both sides of the touch-screen table. As for the Control group of Exp. 1, the probability to select a reward-yielding target was 50% in both the near space (3 proximal rows) and the far space (3 distal rows). Participants were not permitted to communicate verbally with one another.

### Procedure

The procedure was the same as in Exp. 1, except that the reachability-judgment task and the target-selection task were each performed by two participants, facing each other. After having collected written consents and laterality scores, as well as arm length, inter-shoulder distance and eye-level height for both participants, instructions relative to the two tasks were provided by the experimenter. During the reachability-judgment task, participants were seated facing each other with their right hand at rest on the touch-screen table in front of their chest. Their left hand was positioned on the left side of the touch-screen table on a computer keyboard, used to provide reachability estimates. Each of the 31 target locations was presented 4 times, in random order, for a total of 124 stimuli.

Following the first reachability-judgment task (pretest), the mirror was removed, allowing the two participants to see their hands and the touch-screen table. In each block of trials, participants selected, in turn, 6 out of the 32 targets presented at random locations with the aim of collectively maximizing the point count by finding the reward-yielding targets, i.e. those turning green. The two participants performed 34 blocks of 12 manual reaching actions yielding 408 successive target selections (204 for each participant). Following this task, a second reachability-judgment task (posttest) was performed. Finally, a post-experiment debriefing was administered to assess whether participants were aware of the hypotheses tested, which was the case for none of them were. In all, the experimental session lasted approximately 45 minutes.

### Data and analysis

Reachability estimates made for the 31 targets were analysed separately for the pretest and posttest, with all estimates being corrected for actual arm length on a per-participant basis. The boundary of peripersonal space was determined as in Exp. 1, using a maximum likelihood fit based on the second-order derivatives to obtain the logit regression model that best fitted the reachable/unreachable responses. The goodness of fit in logistic regression, the discrimination threshold and reaction time were computed as in Exp. 1. The boundary of peripersonal space, the discrimination threshold and the goodness of fit in logistic regression were analysed using a repeated measures ANOVA on Session (Pretest, Posttest). Reaction time was analysed using a Session (Pretest, Posttest) × Space (Peripersonal, Boundary, Extrapersonal) ANOVA, with repeated measures on both factors.

The target-selection task was analysed as in Exp 1. We computed the location of the targets selected across the manual reaching actions. This was done by plotting the number of times each target location on the touch-screen table was selected relative to the total number of targets (density map, considering each participant’s location), pooling the data from the two proximal, the two central and the two distal rows. Statistical analysis was performed using a Participant location (A, B) × Space (Proximal, Central, Distal) Chi-squared test. In order to precisely compare the performances of participants depending on their location (A, B), we computed the number of times each participant selected a target, as well as the number of successful actions (reward-yielding targets), in the proximal and distal hemi-spaces. Statistical analysis was conducted using a Participant location (A, B) × Block (2 to 34) ANOVA, with repeated measures on the last factor. Finally, we computed the mean amplitude of manual reaching actions to the targets selected in the different blocks and statistically compared the first blocks (2, 3, 4) to the last blocks (32, 33, 34) through a Participants location (A, B) × Block (First, Last) ANOVA, with repeated measures on Block (the first block was in all cases regarded as practice and was not included in the statistical analysis). Three blocks were considered at the beginning and end of the target-selection task in order to match in Exp. 2 the number of manual reaching actions considered in Exp. 1 (respectively 18 and 20).

For all analyses, distribution normality was tested using the Shapiro-Wilk test, and homogeneity of the covariance matrices was tested using the Box's M test. In case of lack of normality of the data distribution, a non-parametric test was used to compare conditions (Wilcoxon Test for paired samples). In case of violation of the sphericity assumption (i.e., epsilon < 1), Huynh-Feldt adjustments to the p-values were reported. Effect sizes were indexed using partial Eta-squared (η^2^_p_). Post-hoc comparisons were performed using *Tukey's* HSD test (α = .05 for all comparisons).

## Results

### Reachability estimates

In the pretest, the mean perceived boundary of peripersonal space overestimated the actual maximum reachability by 3.91 cm on average, which corresponded to an overerestimation of arm length by 5.40% (average arm length = 72.35 cm). The one-way ANOVA on reachability estimates revealed a significant effect of Session (F(1, 19) = 5.46, p = .01, η^2^_p_ = 0.22). Peripersonal space grew by 3.19 cm in the posttest (7.10 cm) compared to the pretest (3.91 cm). Fourteen of the 20 participants (70%) individually showed the group's directional bias (see individual results, Fig 2). Concerning the goodness of fit in logistic regressions, R coefficient (on average 0.818) was found to be not different in the pretest (0.835) compared to the posttest (0.801; Wilcoxon T = 74.00, p = .25). The discrimination threshold was on average 9.85 cm, and did not change significantly from pretest (8.69 cm) to posttest (11.01 cm; F(1, 19) = 0.51, p = .48).

Analysis of reaction time (606 ms on average, see Table 2) showed an effect of Session, (F(1,19) = 24.86, p < .001, η^2^_p_ = 0.57), with faster reaction time in the posttest (572 ms) than in the pretest (641 ms). There was also an effect of Space, (F(2,38) = 40.83, p < .001, η^2^_p_ = 0.68), with faster reaction time to targets located in peripersonal space (529 ms) than at the boundary of peripersonal space (682 ms, p < .001) or in extrapersonal space (608 ms, p < .001). The two latter reaction times were also different from one another (p < .001), and there was no interaction between Space and Session (F(2,38) = 1.58, p = .22).

**Table 2:** Mean reaction time (SD in brackets) in the reachability-judgment task of Exp 2.

Condition	Session	Peripersonal	Boundary	Extrapersonal
**Social**	Pretest	559 (74)	727 (113)	635 (78)
	Posttest	498 (89)	637 (133)	580 (87)
**Control**	Pretest	653 (151)	811 (147)	777 (154)
	Posttest	572 (155)	697 (191)	662 (160)

### Target-selection task

**Fig 6:**
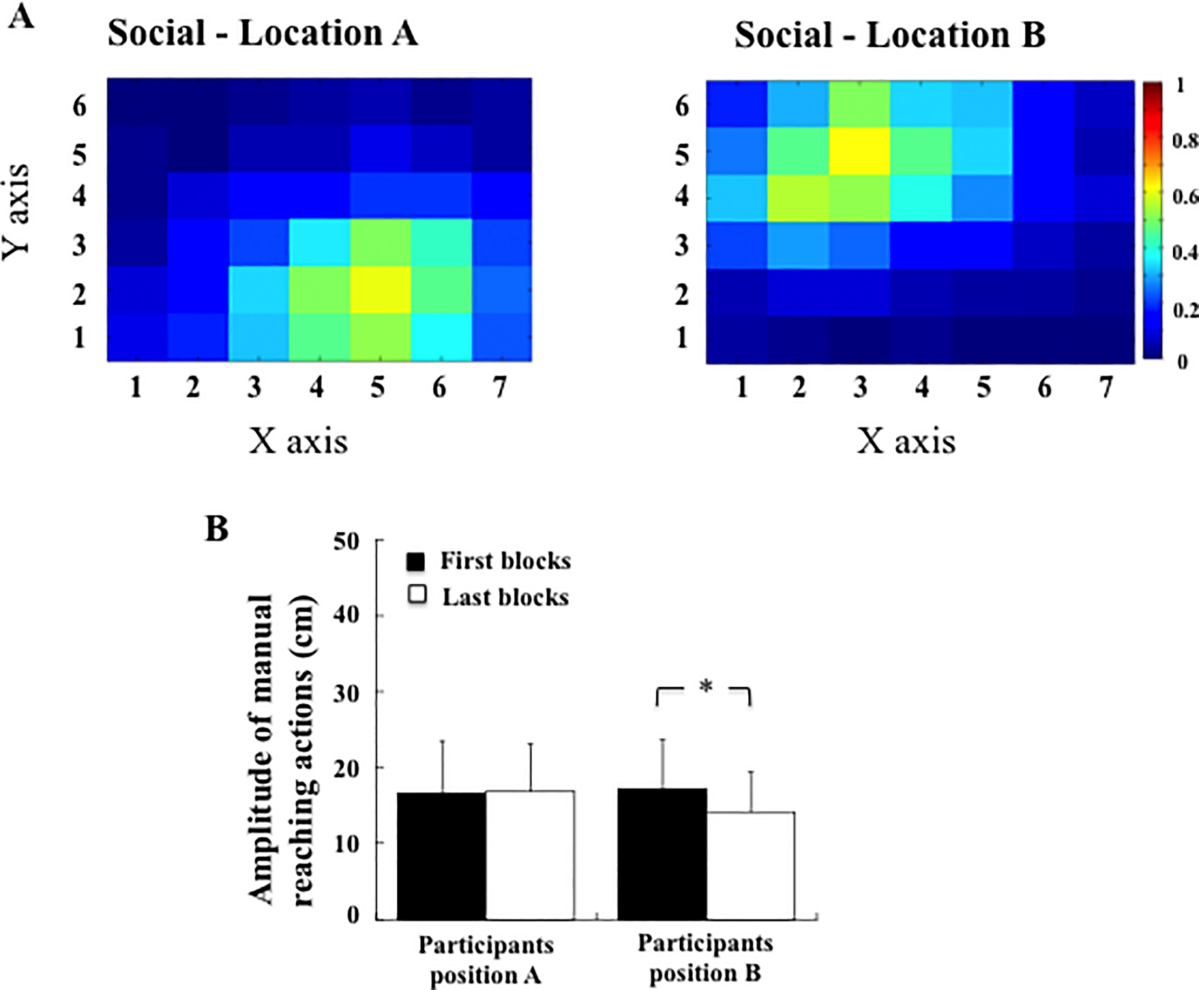
(A) Density map of the targets selected in the target-selection task by all participants across the 408 manual reaching actions in the social experiment. Blue colour indicates infrequent selection; Red colour indicates frequent selection. (B) Mean amplitude of manual reaching actions in the first and last blocks for the participants in position A and position B (Exp. 2). Stars indicate significant differences.

The amplitude of manual reaching actions across the 408 target selections was on average 18.1 cm, the length of the touch-screen table being 50 cm (Location A: 18.5 cm, Location B: 17.6 cm). Comparing the selected targets in the proximal (rows 1 and 2), central (rows 3 and 4) and distal (rows 5 and 6) spaces, we observed that the frequency of selection depended on the participant's location (χ^2^(2) = 1582, p < .001). Among the 2040 targets selected by the participants at each location (6 targets x 34 blocks x 10 participants), participants at location A selected 1197 targets (59%) in their proximal space and 140 targets (7%) in their distal space. Participants at location B selected 1050 targets (51%) in their proximal space and 125 targets (6%) in their distal space. The density map presented in Fig 6A describes the distribution of the targets selected by all participants depending on their location.

When comparing the first blocks to the last blocks (Fig 6B), we found a significant interaction between Block (First, Last) and Location (A, B), F(1, 18) = 5.31, p = .03; η^2^_p_ = 0.23). Local comparisons showed that performances did not differ in the first and last blocks for participants at location A, who selected proximal targets consistently (respectively at 16.6 and 17.1 cm). Participants at location B also kept selecting proximal targets even though nearer in the last than the first blocks (respectively at 16.9 and 14.1 cm, p = 0.03).

**Fig 7:**
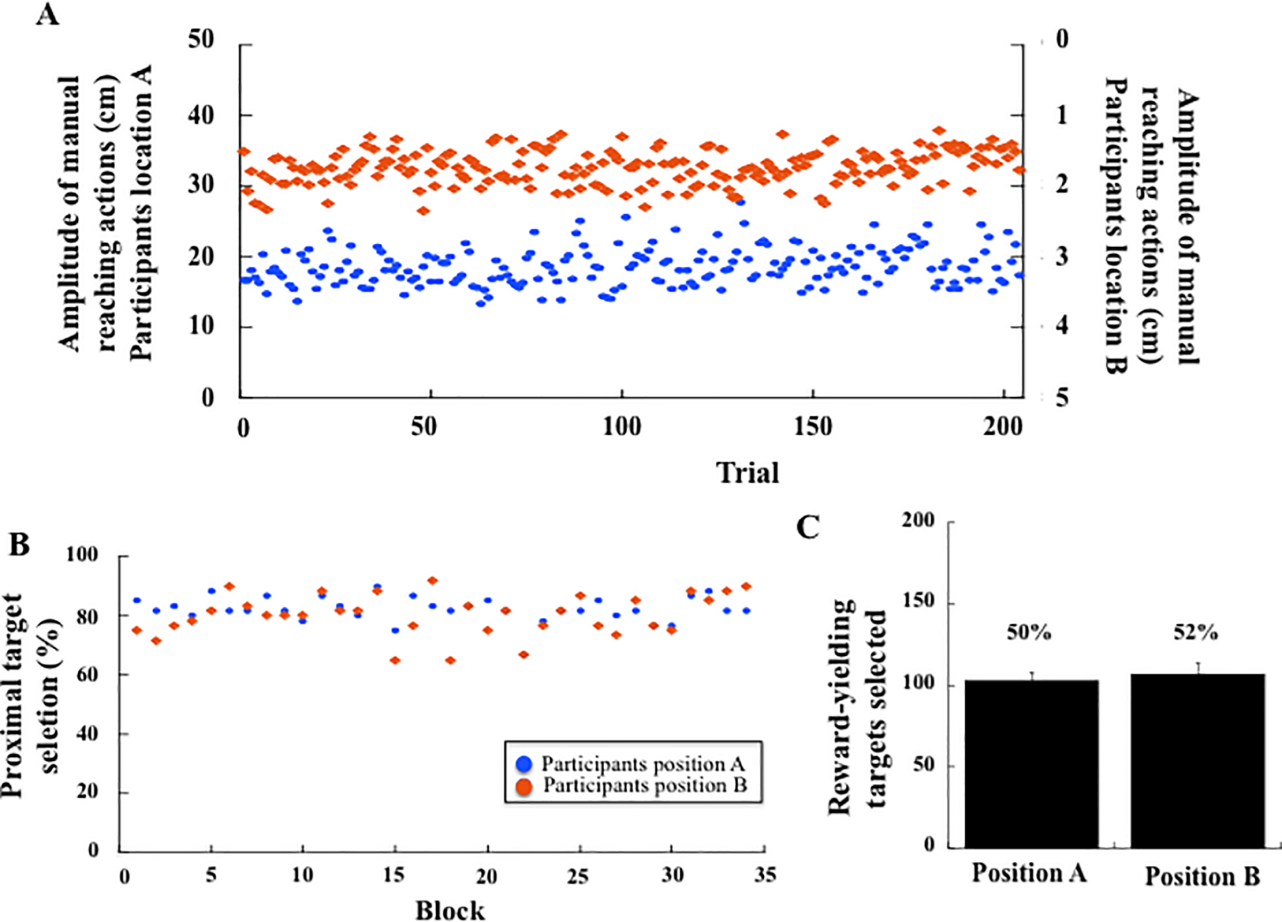
(A) Mean amplitude of manual reaching actions in the target-selection task across the 408 target selections (Exp. 2) for the participants at position A (left y axis) and position B (right y axis). (B) Mean percentage of targets selected in the proximal space (row 1, 2, 3) across the 34 blocks. The performance was not influenced by the participants' position (A, B). (C) Mean number and percentage of reward-yielding targets selected for the participants at position A and position B.

Target selection showed nearly no change across blocks (Fig 7A). Analysis of the number of times targets were selected in the proximal hemi-space (rows 1, 2, 3 for participant’s location A and 4, 5 6 for participant’s location B) and in the distal hemi-space (rows 4, 5, 6 for participant’s location A and 1, 2, 3 for participant’s location B) showed no significant change across blocks (F(33, 594) = 1.47, p = 0.05, Fig 7B). On average, participants selected 81% of targets in the proximal space (Position A: 82%, Position B: 80%), and these values were stable from the first to the last blocks (respectively 78% and 85%). Furthermore, there was no effect of Participants' location (F(1,18) = 0.16, p = .070), and no Block × Participants location interaction (F(33, 594) = 0.55, p = .98). As a consequence, and in agreement with the design of the experiment, we observed that the percentage of reward-yielding targets selected did not differ depending on the position of the participants (position A: 50% and position B: 52%, F(1, 18) = 1.85, p = .19, Fig 7C).

## Control experiment

In order to exclude the effect of the simple presence of a confederate on the change of peripersonal space representation in a social context, we replicated Exp. 2 but with one participant performing the target selection task while facing a passive confederate. The apparatus, the method and the procedure were the same as in Exp. 2, except for the two following items. First, both participants performed the reachability-judgment task, but during the target-selection task only one participant (the actor) selected the targets, while the other one was simply observing (the observer). Second, the actor selected 12 out of the 32 targets in each of the 34 blocks of trials. The spatial distribution of reward-yielding targets was 50% in both the proximal and the distal spaces. Data recording and analysis were performed as in Exp. 2., but only the actor’s performances were taken into account in both the reachability judgment task and the target selection task. For this purpose we recruited 20 new couples of right-handed (mean laterality score: 0.76, SD: 0.18) participants (aged 18–30 years, mean age: 20.64, SD: 2.87), who gave written informed consent prior to inclusion in the experiment.

Concerning the *reachability judgment task*, the mean perceived boundary of peripersonal space was not different in the pretest (6.96 cm) and in the posttest (7.32 cm; F(1, 19) = 0.09, p = .76), which corresponded to an overestimation of arm length (on average 71.68 cm) of 9.96% (Fig 2A). Individual results revealed a balanced distribution between participants showing a slight increase or decrease of peripersonal space (Fig 2B). Concerning the goodness of fit in logistic regressions, R coefficient (on average 0.793) was found to be not different in the pretest (0.778) compared to the posttest (0.809; Wilcoxon T = 95.5, p = .72). The discrimination threshold was on average 8.42 cm, and did not change significantly from pretest (10.21 cm) to posttest (6.63 cm; F(1, 19) = 2.65, p = .12). Analysis of reaction time (695 ms on average, see Table 1) showed an effect of Session, (F(1,19) = 41.30, p < .001, η^2^_p_ = 0.68), with faster reaction time in the posttest (644 ms) than in the pretest (746 ms). There was also an effect of Space, (F(2,38) = 29.86, p < .001, η^2^_p_ = 0.61), with faster reaction time to targets located in peripersonal space (613 ms) than at the boundary of peripersonal space (754 ms, p < .001) or in extrapersonal space (718 ms, p < .001). The two latter reaction times were not different (p = .14), and there was also no interaction between Space and Session (F(2,38) = 0.68, p = .51).

**Fig 8:**
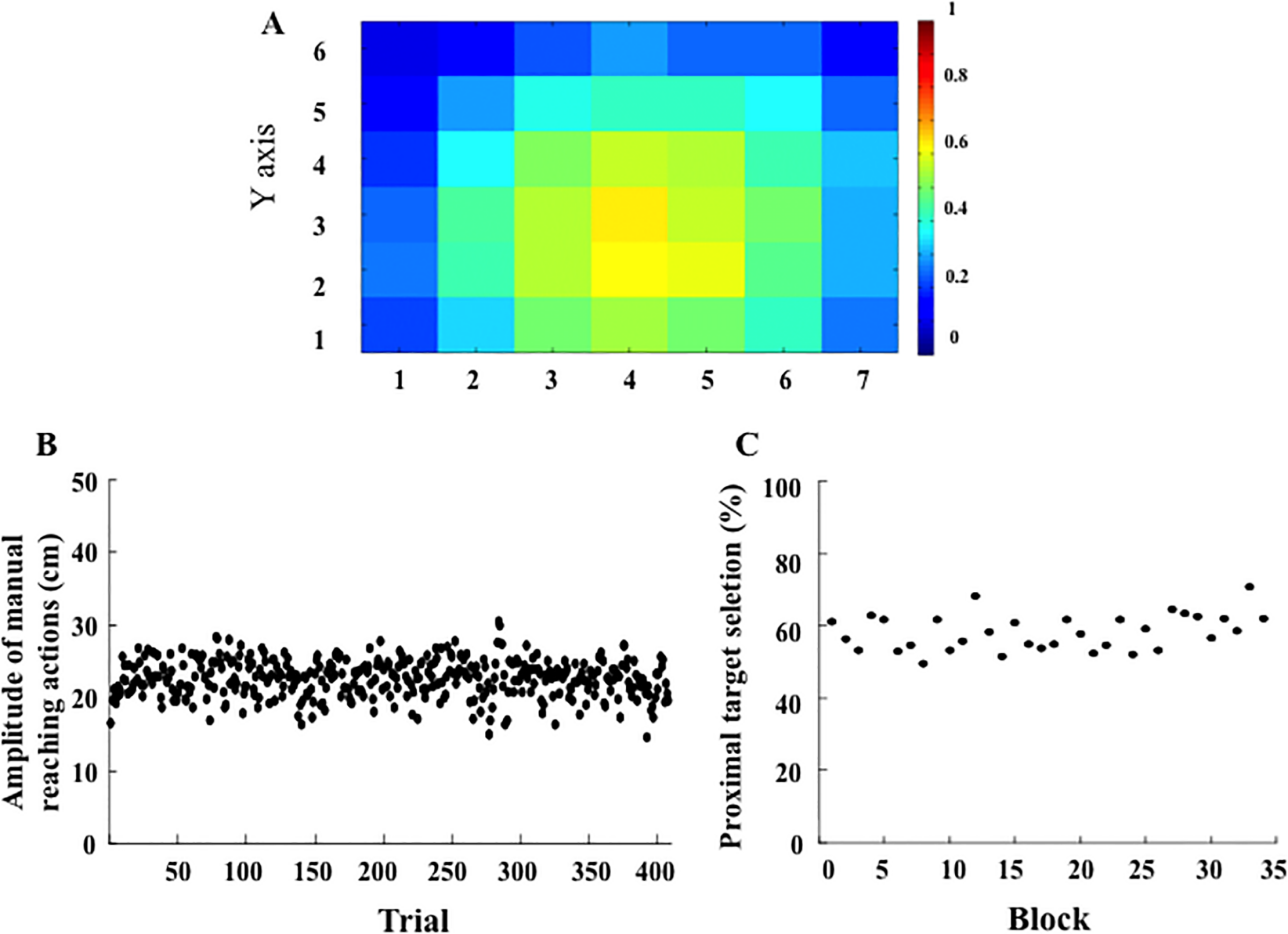
(A) Density map of the targets selected in the target-selection task by all participants across the 408 manual reaching actions in the control experiment. Blue colour indicates infrequent selection; Red colour indicates frequent selection. (B) Mean amplitude of manual reaching actions across the 408 target selections. (C) Mean percentage of targets selected in the proximal space (row 1, 2, 3) across the 34 blocks.

Concerning the *target selection task*, the amplitude of manual reaching actions across the 408 target selections was on average 22.68 cm. Among the 8160 targets selected by all participants (12 targets x 34 blocks x 20 participants), 3066 targets (38%) were located in their proximal space and 1905 targets (23%) were located in their distal space (χ^2^(2) = 592.51, p < .001; Fig 8A). Although the amplitude of reaching actions showed no clear variation across the blocks (Fig 8B), it differed slightly in the first blocks (23.28 cm) compared to the last blocks (21.14 cm; F(1, 19) = 4.99, p = .04, η^2^_p_ = 0.21). The number of times the targets were selected in the proximal hemi-space (rows 1, 2, 3, on average 58.24%) and in the distal hemi-space (rows 4, 5, 6, on average 41.76%) did not differ significantly across blocks (according to the chi-square approximation of the Friedman test: χ^2^ (33) = 47.06, p = .053, Fig 8C). As a consequence, and in agreement with the design of the experiment, we observed that the percentage of reward-yielding targets selected was 52%.

## Discussion

The aim of Exp. 2 was to assess the effect of performing a target-selection task in a social context on manual reaching actions and peripersonal space. The a priori probability of selecting a reward-yielding target for the two interacting participants was distributed homogeneously across the workspace, i.e. 50% in both the distal and proximal spaces. One important result of this experiment is that, despite the objective remained the same in Exp. 2 and Exp. 1, the target-selection task was performed differently in the social and non-social context. As revealed by the density maps, in the social context participants selected predominantly targets in their proximal space and on the right-hand side (55% on average) instead of targets in the distal space (6% on average). This is compatible with performing the selection task using the right arm (biomechanical constraint) and avoiding invading the confederate’s peripersonal space (social constraint). As a consequence, the average amplitude of manual reaching actions towards the selected targets was short and consistent across the participants (18.1 cm on average), whatever their location. Furthermore, the strategy used to select the targets did not change across blocks, with an average of 81% of the targets selected being in the proximal space. Overall, these findings support the previous observation that people tend to assign specific regions of the workspace to one another in cooperative motor tasks—even if only implicitly—and thus adapt their behaviour to fit the social context [69–70]. However, this typical behaviour was observed when all participants were cooperative, not when one of the confederate was simply observing.

Another important outcome of the present experiment is that, despite the manual reaching actions having constant average amplitude throughout the target-selection task, most participants increased their peripersonal space after having completed this task in cooperation (3.19 cm on average). This effect of the target-selection task was surprising considering that the targets were predominantly selected in the proximal space, which was found to induce a reduction, not an extension, of peripersonal space (see Exp. 1). The increase of peripersonal space was not accompanied with a change of the discrimination threshold, suggesting that the reachability-judgment task was equally difficult after and before having performed the target-selection task. Reaction time was nonetheless 70 ms shorter in the posttest, suggesting a positive practice effect. One possible interpretation for the effect of the target-selection task on the representation of peripersonal space is that the presence of a confederate at close proximity, while performing the target selection task, induced an expansion of peripersonal space. However, the control task indicates that this interpretation is hardly plausible, since no change of peripersonal space was observed when the participants performed the target selection task while facing a passive confederate, confirming previous observations of the weak effect of the presence of a confederate on spatial tasks in a non-interactive social situation [71–72]. Likewise, the change of the representation of peripersonal space cannot be simply associated with successful manual reaching actions since the participants chiefly selected targets in the proximal space despite the equidistribution of reward-yielding targets across the workspace. In a non-social context, selecting targets predominantly in the proximal space was found to produce shrinkage of peripersonal space, not its expansion (see Exp. 1). The most likely explanation for the observed expansion of the representation of peripersonal space in the cooperative condition is that it resulted from the positive outcome of participants’ own actions combined with that registered following the conspecifics' actions (theoretically, 50% were expected to be successful). Considering the cooperative context of the task, one may assume that all reward-yielding targets selected by the conspecific were also appropriated as positive outcomes by the participants, since the points gained by the two participants across the manual reaching actions contributed to a shared total. This interpretation is supported by the significant relation found between the rate of reward-yielding targets obtained in the distal (or proximal) space and the increase (or decrease) of peripersonal space. Such relation was not found when analysing the relation between the change of movement amplitude and the change of peripersonal space. Accordingly, successful manual reaching actions performed by the confederate could have had the effect of extending the participants’ own representation of peripersonal space in the direction of the confederate’s location.

As a whole, the results show that a cooperative social context modifies the representation of peripersonal space, confirming previous findings [71–72]. Moreover, the present experiment extends these findings by showing that the effect of social context on the representation of peripersonal space depends on the outcome not only of the self-executed actions but also those performed by others in a social interaction context.

## General discussion

The aim of the present study was to evaluate the effect of a target-selection task on manual reaching actions and peripersonal space representation when the spatial distribution of the reward-yielding targets was manipulated and the target-selection task was performed in either an individual or social context. The main outcome of this study was that the spatial distribution of the reward-yielding targets altered both manual reaching actions and the representation of peripersonal space, although differently in the individual and social context. The decrease in amplitude of manual reaching actions resulting from the biased distribution of reward-yielding targets was associated with a reduction of peripersonal space. By contrast, the decrease in amplitude of manual reaching actions resulting the social interaction context was associated with an increase of peripersonal space, which was not due to the mere presence of a confederate (control experiment). In the social context, the extent of peripersonal space seemed thus to depend both on the successful manual reaching actions performed by the participants, and those observed in the confederate seated across the table. We may thus assume that the observed manual reaching actions were processed by participants from a first-person perspective [67, 73], integrating the outcomes of the observed actions, as if they were performed by the observer in agreement with the mirror system hypothesis [74–75]. In support of this view, mirror neurons in monkey studies were found to be space-selective and also sensitive to the viewpoint during live-action observation [76]. We may thus speculate that, in a social context, participants’ representation of peripersonal space extended so as to overlap or merge with the peripersonal space of the (successful) facing conspecific. The process of overlapping or merging co-actors’ representations of peripersonal space is thought to create a shared action space supporting key computations of social interactions and joint actions [77].

Socially-induced extensions of peripersonal space have been observed in situations implying inter-individual cooperation. For instance, Costantini, Ambrosini, Sinigaglia and Gallese [78] showed that observing a conspecific using a tool produced an extension of the representation of the observer's peripersonal space. The same group also showed that neglect syndrome in right-hemisphere stroke patient can be shifted from near to far space by simply observing the clinician using a tool [64]. In the same vein, an increase of peripersonal space was reported in a situation where participants played a cooperative economics game with a partner before performing a tactile detection task on their face in the presence of concurrent approaching task-irrelevant sounds [72]. Multisensory integration of information, a proxy used to estimate the size of peripersonal space, was observed at a larger distance after having played the cooperative game than before. The present study extends these findings by showing, for the first time, that the expansion of peripersonal space depends on the outcome associated both to self-executed actions but also to those performed by a confederate in interactive contexts.

Although peripersonal space was altered by the spatial distribution of reward-yielding targets in both the isolated and the social context, the manual actions underwent more reorganization in the former than in the latter context. We assume that adaptation of the manual reaching actions involved reward-based learning mechanisms contributing to the formation of new sensorimotor associations, linking visual stimuli to action selection mechanisms [79]. Accordingly, performing a particular action in a multiple choice situation depends not only on the properties of the present stimuli in relation to the motor system, by weighing the likelihood and costs of each potential action [80], but also on the benefits of performing that particular action [81]. Parameters modulating perceptual selection and action selection thus need to be integrated within a common representation [82].

The present study provides new insight in this respect, extending the results reported in studies of visual attention [53, 83–85], gaze control [39, 86–87] and manual actions [81], in which it was found that the visual system gives priority to intrinsically valuable stimuli, i.e. reward-contingent stimuli. In particular, shorter reaction times and larger peak velocities were observed for actions performed towards the high-valued stimuli [88–89]. Therefore, in the present study, we may consider that participants implicitly, but reliably, learned which target locations optimised the probability of reward. This modified both the range of potential actions considered and the representation of peripersonal space. However, although reinforcement learning theory explains how reward-predicting events are assigned a higher value and become targets of behaviour [90–93], the present study demonstrated that a higher value assigned to a visual stimulus can emerge from self-generated actions as well as from observed actions performed by others.

With respect to the neural basis of action-target association, we hypothesise that a broad neural circuit involved in reward processing contributed to the selection of visual information and motor responses in our target-selection task. In particular, the response properties of dopamine neurons have been found to reflect key mechanisms for reinforcement learning, such as the positive or negative consequences of perceptual and motor decisions, as well as the difference between expected and actual reward outcomes [94]. Beyond the midbrain, dopaminergic areas sensitive to the hedonic value of stimuli in the environment, many subcortical (striatum, amygdala, superior colliculus) and cortical (prefrontal, cingulate, parietal, inferotemporal cortex) structures involved in high-level sensory and/or motor integration receive information related to reward [95–97]. As a result, these cortical regions respond specifically to conditioned stimuli and responses that predict future reward [98]. In particular, the ventromedial prefrontal cortex (vmPFC), the orbitofrontal cortex (OFC) and the anterior cingulate cortex (ACC) are thought to contain key neuronal mechanisms for processing value information related to the selection of stimuli and behaviour [99]. The OFC and ACC are both implicated in reinforcement-guided decision-making, error monitoring, and behavioural adaptation in response to changing circumstances [99–100]. The ACC is furthermore critical when decisions are based on the association between reward and action or the determination of the action with the highest reward potential [101]. The vmPFC preferentially guides action selection in a multi-choice context depending on previous evaluation of objects' value [92]. These cortical regions probably influence the dorsal premotor cortex, which contributes to the selection of the most appropriate actions from among candidate actions [80]. Another crucial component of this network is the striatum where affective, cognitive, and motor information converge [102–103] to influence learning and decision-making [104]. Interestingly, these cortical areas have also been found to be sensitive to rewards in social contexts [105]. Neurophysiological underpinnings of the reward system thus suggest that learning of sensorimotor associations, decision making and response selection share common neural substrates [79, 82, 95, 106] and are influenced by the value of stimuli in individual as well as in social contexts [107].

The changes in peripersonal space in relation to the distribution of the reward-yielding targets observed in the present study might then be conceived as an influence of the reward network on the brain network associated with the representation of peripersonal space. The representation of peripersonal space involves brain regions with spatial and motor functions in the parietal cortex, the premotor cortex and the prefrontal regions [3, 26]. It is thus conceivable that the reward network modulates the activity of these brain regions so as to adjust the contours of peripersonal space depending on the value of stimuli and the social context. In agreement with this, monkey studies have shown that parietal cortical activity (anterior-medial IPS) shows a marked preference for reward-yielding stimuli that are within reach. However, in cases of conflict of interest between monkeys, the submissive monkey's parietal neurons lose their marked preference for certain stimuli that usually trigger self-generated movements [47]. In a related study, Fujii et al. [48] found a concomitant modulation of the neuronal activity in the parietal and pre-frontal cortices, suggesting that these two regions are a part of a larger social-cognitive brain network. According to these authors, the parietal cortex is thought to represent environmental spatial information linked with the self’s body image and action system, whereas the pre-frontal cortex is thought to integrate multimodal sensory and social information in an abstract workspace to maintain a constantly updated representation of the physical and social environment in the service of response selection.

In conclusion, the present study revealed that reward-dependent modulation of objects value in the environment modified both stimulus-action binding and the representation of peripersonal space, but differently in an individual vs a social context. In particular, we found that peripersonal space increased when reward-yielding stimuli were selected in the distal instead of the proximal space, or when they were selected in the distal space by a conspecific in a social interaction context. The representation of peripersonal space must thus be viewed as an integration of body properties and the value of the objects in the environment, including also the outcome of observed actions performed by a confederate in a cooperative social context. The findings of the present study provide new avenues for research. It would for instance be interesting to investigate how the non-homogeneous spatial distributions of reward-yielding targets in a social context modulates the organisation of the workspace and the representation of peripersonal space. It would be also interesting to investigate the effect of manipulating the social context by implementing a competitive instead of a cooperative condition in the target-selection task.
